# Zika virus induces neuronal and vascular degeneration in developing mouse retina

**DOI:** 10.1186/s40478-021-01195-6

**Published:** 2021-05-25

**Authors:** Yi Li, Shuizhen Shi, Fan Xia, Chao Shan, Yonju Ha, Jing Zou, Awadalkareem Adam, Ming Zhang, Tian Wang, Hua Liu, Pei-Yong Shi, Wenbo Zhang

**Affiliations:** 1 Department of Ophthalmology and Visual Sciences, University of Texas Medical Branch, 301 University Boulevard, Galveston, TX 77555-0144 USA; 2 Department of Biochemistry and Molecular Biology, University of Texas Medical Branch, 301 University Boulevard, Galveston, TX 77555-0144 USA; 3 Department of Microbiology and Immunology, University of Texas Medical Branch, Galveston, TX USA; 4 Department of Cellular Biology and Anatomy, Augusta University, Augusta, GA USA; 5 Sealy Center for Vector-Borne and Zoonotic Diseases, University of Texas Medical Branch, Galveston, TX 77555 USA; 6 Institute for Human Infections and Immunity, University of Texas Medical Branch, Galveston, TX 77555 USA; 7 Departments of Neuroscience, Cell Biology and Anatomy, University of Texas Medical Branch, Galveston, TX USA

**Keywords:** Zika virus, Retina, Neuronal degeneration, Vascular degeneration, Inflammation, Endoplasmic reticulum stress, Drug efficacy, Vaccine safety

## Abstract

Zika virus (ZIKV), a mosquito-borne flavivirus, can cause severe eye disease and even blindness in newborns. However, ZIKV-induced retinal lesions have not been studied in a comprehensive way, mechanisms of ZIKV-induced retinal abnormalities are unknown, and no therapeutic intervention is available to treat or minimize the degree of vision loss in patients. Here, we developed a novel mouse model of ZIKV infection to evaluate its impact on retinal structure. ZIKV (20 plaque-forming units) was inoculated into neonatal wild type C57BL/6J mice at postnatal day (P) 0 subcutaneously. Retinas of infected mice and age-matched controls were collected at various ages, and retinal structural alterations were analyzed. We found that ZIKV induced progressive neuronal and vascular damage and retinal inflammation starting from P8. ZIKV-infected retina exhibited dramatically decreased thickness with loss of neurons, initial neovascular tufts followed by vessel dilation and degeneration, increased microglia and leukocyte recruitment and activation, degeneration of astrocyte network and gliosis. The above changes may involve inflammation and endoplasmic reticulum stress-mediated cell apoptosis and necroptosis. Moreover, we evaluated the efficacy of preclinical drugs and the safety of ZIKV vaccine candidate in this mouse model. We found that ZIKV-induced retinal abnormalities could be blocked by a selective flavivirus inhibitor NITD008 and a live-attenuated ZIKV vaccine candidate could potentially induce retinal abnormalities. Overall, we established a novel mouse model and provide a direct causative link between ZIKV and retinal lesion in vivo, which warrants further investigation of the underlying mechanisms of ZIKV-induced retinopathy and the development of effective therapeutics.

**Supplementary Information:**

The online version contains supplementary material available at 10.1186/s40478-021-01195-6.

## Introduction

Zika virus (ZIKV) is an enveloped and spherical flavivirus which is transmitted by *Aedes* mosquitoes [15]. The virus was initially isolated from a rhesus monkey in the Zika Forest of Uganda in 1947 [8], and has caused outbreaks in Asia, the Pacific island and more recently in South and Central America [3, 4, 24], followed by a rapid spread to other countries during 2015–2016 including autochthonous transmissions in Florida and Texas in the United States [11]. Although symptomatic infection in humans results in mostly a mild and self-limiting febrile disease, it has also been linked to a neurological autoimmune disorder Guillain-Barré syndrome in adults and microcephaly in fetuses and infants born to infected mothers during pregnancy [3, 15, 34]. In addition to neuronal damage in the brain, infants with congenital ZIKV infection are associated with a high rate of ocular abnormalities in which the most common lesions are retinal lesions, chorioretinal atrophy and optic nerve abnormalities. Occasionally calcification in the retina and lens are also noted [5]. Mechanisms of ZIKV-induced retinal abnormalities are unknown and no therapeutic intervention is available to treat or minimize the degree of vision loss in patients.

Studies of pathology related to ZIKV infection in human subjects have been limited since most of the infected individuals are asymptomatic or mildly symptomatic; and only 4–8% fetuses or infants from pregnant women with confirmed ZIKV infection exhibit ZIKV-associated birth defects [28, 38]. Therefore animal models are critical for investigation of ZIKV-induced pathology. Mouse models have been widely used in biomedical research due to low-cost maintenance, a short reproductive cycle and easy genetic modification. Because ZIKV does not replicate efficiently in adult wild type (WT) mice, blocking type I interferon receptor (IFNAR1) by gene deletion or blocking antibody is necessary to overcome infection barrier [1]. However, no apparent retinal pathology or abnormality was observed in ZIKV-infected adult mice with IFNAR blockade or in IFNAR heterozygous fetuses from ZIKV-infected IFNAR deficient (IFNAR1^−/−^) dam despite the presence of viremia [16] and moderate loss of retinal ganglion cells (RGCs) and cones at 60–70 days after infecting 3-week-old A129 IFNAR1^−/−^ mice [36]. Moreover, blockade of IFN pathway causes immune deficiency which may compromise the understanding of pathological changes upon ZIKV infection. Chorioretinitis and retinal cell death were found in adult WT mice receiving injections of a substantial amount of ZIKV into vitreous or aqueous humor [30, 31]. Nevertheless, intravitreal injection or intra-aqueous injection is not a disease-relevant route for ZIKV transmission to the retina. Two studies show intra-amniotic injection of ZIKV into C57BL/6 WT mice at embryonic day 13.5 (E13.5) or E15 allow infected mice to grow into puberty and recapitulate several symptoms of clinical congenital Zika syndrome including severe retinal neuronal degeneration [6, 29]. The limitation of intra-amniotic injection is that this procedure requires surgery and special skills and the maternal immune response and placental insufficiency may affect neural development, making it complicated to interpret the data.

Mouse retinal development is initiated at approximately E12. It continues during the first 3 weeks after birth in a process very similar to human retinal development during the third trimester of pregnancy [9]. At birth (postnatal day (P) 0), mouse retina consists of a multitude of neuroblasts (progenitors) and some differentiated RGCs, amacrine cells and horizontal cells. From P0 to P8, retinal development continues, including the formation of bipolar cells, photoreceptors (rods and cones) and a single glial cell type (Müller cells) by neuroblasts in a temporally ordered sequence [18], the formation of each retinal layers, and the maturation of neuronal synapses [9]. Coincident with the final stages of neuronal differentiation, the eyes are open and the vision process is initiated at P14. Mouse retinal vascularization begins at P0 and completes by P21 [9]. Taking these advantages, we established a novel mouse model using postnatally developing mouse retina to study retinal abnormalities associated with ZIKV, provided a causative link between ZIKV and retinal lesion in vivo, and demonstrated its use to test the efficacy and safety of therapeutic interventions.

## Materials and methods

### Mice

C57BL/6J mouse colony was purchased from Jackson Laboratory (Bar Harbor, ME) and maintained in the animal facility at the University of Texas Medical Branch. Mice were housed under standard conditions of 12:12 light/dark cycle with food and water available ad libitum. Animal protocols were approved by the Institutional Animal Care and Use Committee. All experimental procedures and use of animals were performed in accordance with the Association for Research in Vision and Ophthalmology Statement for the Use of Animals in Ophthalmic and Vision Research.

### ZIKV infection and inhibitor treatment

ZIKV Cambodian strain FSS13025 that is phylogenetically closely related to those circulating in the Americas was produced using an infectious complementary DNA (cDNA) clone as described previously [26]. A live-attenuated ZIKV vaccine candidate that contains a 10-nucleotide deletion in the 3' untranslated region of the ZIKV genome (10-del ZIKV) was developed and produced using the FSS13025 infectious clone [25]. ZIKV (20 PFU in 10 μl PBS) or 10-del ZIKV (20 or 200 or 2000 PFU in 10 μl PBS) was injected into neonatal mice at Postnatal day (P) 0 subcutaneously. Sofosbuvir (SOF) (80 mg/kg) was injected right after ZIKV injection once every day until day 13 (P0–P7: subcutaneously; P8-P13: intraperitoneally). NITD008 (5 mg/kg) was injected right after ZIKV injection and once every other day until day 12 (P0–P6: subcutaneously; P8–P12: intraperitoneally). Vehicle (PBS with 1% DMSO) was injected into control mice. All infected mice were observed every day. The eyeballs were harvested at P5, P8, P11, P14 and P21.

### Immunofluorescence staining of retinal sections

Eyeballs were fixed in 4% paraformaldehyde (PFA) for 60 min, equilibrated in 30% sucrose overnight, and embedded in OCT compound. Cryosections (10 μm) were cut through the optic nerve, post-fixed with 4% PFA for 10 min, rinsed with PBS, permeabilized with PBS containing 0.1% Triton X-100 for 15 min at room temperature, and blocked with PowerBlock (BiogenX, San Ramon, CA) for 1 h at room temperature. Next, sections were probed with primary antibodies against calbindin (1:1000, ab82812, Abcam, Cambridge, MA), CD31 (1:1000. 553369, BD Biosciences, San Jose, CA), cleaved-caspase3 (1:400, 9661, Cell signaling, Danvers, MA), cone-arrestin (1:5000, AB15282, MilliporeSigma, Burlington, MA), Dab1 (1:3000, 18936, Rockland, Pottstown, PA), GFAP (1:500, Z033401-2, Agilent Technologies, Santa Clara, CA), glutamine synthetase (1:1000, MAB302, MilliporeSigma), PKCα (1:500, P5704, MilliporeSigma), pRIP3 (1:1000, 205421, Abcam), rhodopsin (1:2000, sc-57432, Santa Cruz Biotechnology, Santa Cruz, CA) and ZIKV antibody (1:500, NBP2-52666, Novus Biologicals, Littleton, CO) overnight at 4 °C. After washing with PBS, sections were incubated with appropriate secondary antibodies for 1 h and mounted with medium containing DAPI (Abcam) or nuclei were counterstained with propidium iodide (PI; ThermoFisher Scientific, Waltham, MA). Images were taken with confocal microscopy (LSM 800, Carl Zeiss, Inc., Thornwood, NY).

### Immunostaining of retinal whole-mounts

Eyes were enucleated and fixed in 4% PFA at 4 °C overnight. Next day, retinas were dissected, washed with PBS, blocked and permeabilized with PBS containing 5% normal goat serum and 0.3% Triton X-100 for 3 h at room temperature. Retinas were then incubated with isolectin B4 (ThermoFisher Scientific) or primary antibodies against Iba1 (1:200, 019–19741, FUJIFILM Wako Chemicals, Richmond, VA), Tuj1 (1:400, 801202, BioLegend, San Diego, CA), RBPMs (1:200, ABN1376 (guinea pig) or ABN1362 (rabbit), MilliporeSigma), CD31 (1:200), CD45 (1:400, 550539, BD Biosciences) and GFAP (1:500) overnight at 4 °C. Subsequently, retinas were washed with PBS and then incubated with appropriate secondary antibodies at 4 °C for 4 h. Finally, retinas were mounted with aqua-mount medium (ThermoFisher Scientific), and images were captured with confocal microscopy.

### Hematoxylin and Eosin (H&E) staining

After fixation, retinal frozen sections were immersed into hematoxylin for 5 min followed by washing in double distilled water (ddH_2_O). Slides were then dipped in 0.5% Eosin for 45 s, washed in ddH_2_O, dehydrated in graded ethanol and vitrified by xylene. Slides were mounted with Permount mounting medium (ThermoFisher Scientific). Images were captured by a digital camera in a bright field microscope (Leica Camera Inc., Allendale, NJ) for retinal structure.

### Real-time quantitative PCR

Retinas were collected at P5 or P8 from control and ZIKV-infected mice. Total retinal mRNA was isolated using miRNeasy Mini Kit (Qiagen, Germantown, MD), quantified using NanoDrop (ThermoFisher Scientific), and converted to cDNA using High-Capacity cDNA Reverse Transcription Kit (ThermoFisher Scientific). Quantitative PCR was performed with SYBR Green Master Mix (Applied Biosystems, Waltham, MA) using a PCR system (StepOnePlus; Applied Biosystems). Primer sequences for mouse transcripts were as follows: Hprt For-5′-GAA AGA CTT GCT CGA GAT GTC ATG-3′; Hprt Rev-5′-CAC ACA GAG GGC CAC AAT GT-3′; ZIKV prM gene For-5′-GAG AGC GAG GAA CAT CCA GAC T-3′; ZIKV prM gene Rev-5′-CCT GAA GTT CCT GCT GGG TAG T-3′. Inflammatory genes: CXCL10 For-5′-GGA CGG TCC GCT GCA A-3′; CXCL10 Rev-5′-CCC TAT GGC CCT CAT TCT CA-3′; MCP1 For-5′-GGC TCA GCC AGA TGC AGT TAA-3′; MCP1 Rev-5′-CCT ACT CAT TGG GAT CAT CTT GCT-3; ICAM-1 For-5′-CAG TCC GCT GTG CTT TGA GA-3′; ICAM-1Rev-5′-CGG AAA CGA ATA CAC GGT GAT-3′; IL-1β For-5′-AGT TGA CGG ACC CCA AAA GA-3′; Il-1β Rev-5′-GGA CAG CCC AGG TCA AAG G-3′; IL-6 For-5′-CCA CGG CCT TCC CTA CTT C-3′; IL-6 Rev-5′-TTG GGA GTG GTA TCC TCT GTG A-3′; iNos For-5′-GGC AGC CTG TGA GAC CTT TG-3′; iNos Rev-5′-TGC ATT GGA AGT GAA GCG TTT-3′; TNFα For-5′-GGT CCC CAA AGG GAT GAG AA-3′; TNFα Rev-5′-TGA GGG TCT GGG CCA TAG AA-3′; VCAM-1 For-5′-ACA AGT CTA CAT CTC TCC CAG GAA TAC-3′; VCAM-1 Rev-5′-CAC AGC ACC ACC CTC TTG AA-3′. ER stress genes: GRP78 For-5′-ACT TGG GGA CCA CCT ATT CCT-3′; GRP78 Rev-5′-ATC GCC AAT CAG ACG CTC C-3′; XBP1s For-5′-TGC TGA GTC CGC AGC AGG TG-3′; XBP1s Rev-5′-GCT GGC AGG CTC TGG GGA AG-3′; ATF4 For-5′-TCC TGA ACA GCG AAG TGT TG-3′; ATF4 Rev-5′-ACC CAT GAG GTT TCA AGT GC-3′; CHOP For-5′-CTG GAA GCC TGG TAT GAG GAT-3′; CHOP Rev-5′-CAG GGT CAA GAG TAG TGA AGG T-3′; ATF6 For-5′-TGC CTT GGG AGT CAG ACC TAT-3′; ATF6 Rev-5′-GCT GAG TTG AAG AAC ACG AGT C-3′. Data were normalized to internal control Hprt and the fold difference in different transcripts was calculated by the ΔΔCT method.

### Fluorescence in situ hybridization (FISH) using RNAscope technology

Eyeballs were fixed in 4% PFA for 60 min, equilibrated in 30% sucrose overnight, and embedded in OCT compound. Cryosections (10 μm) were cut through the optic nerve. Then retinal sections were boiled in the target retrieval reagent (Cat #322000, Advanced Cell Diagnostics, Hayward, CA); and protease digestion was performed using protease 3 (Cat #322337, Advanced Cell Diagnostics). Next, the tissue slides were immediately rinsed with water and incubated with Zika probe (Cat #467771, Advanced Cell Diagnostics) targeting JN860885.1, and then hybridized with the signal amplification reagents of RNAscope Fluorescent Multiplex detection kit (Advanced Cell Diagnostics) following the manufacturer's instructions. At the last step, sections were counterstained with DAPI to label nuclei, and images were taken by confocal microscopy.

### Statistical analysis

Statistical analysis was conducted using GraphPad Prism program (GraphPad Software, Version 8.0, La Jolla, CA). Results were presented as mean ± standard error of mean (SEM) and analyzed by Student's t-test. A *P* value < 0.05 was considered statistically significant.

## Results

### ZIKV infection induces retinal neuronal loss and glial disruption and activation

**Fig. 1:**
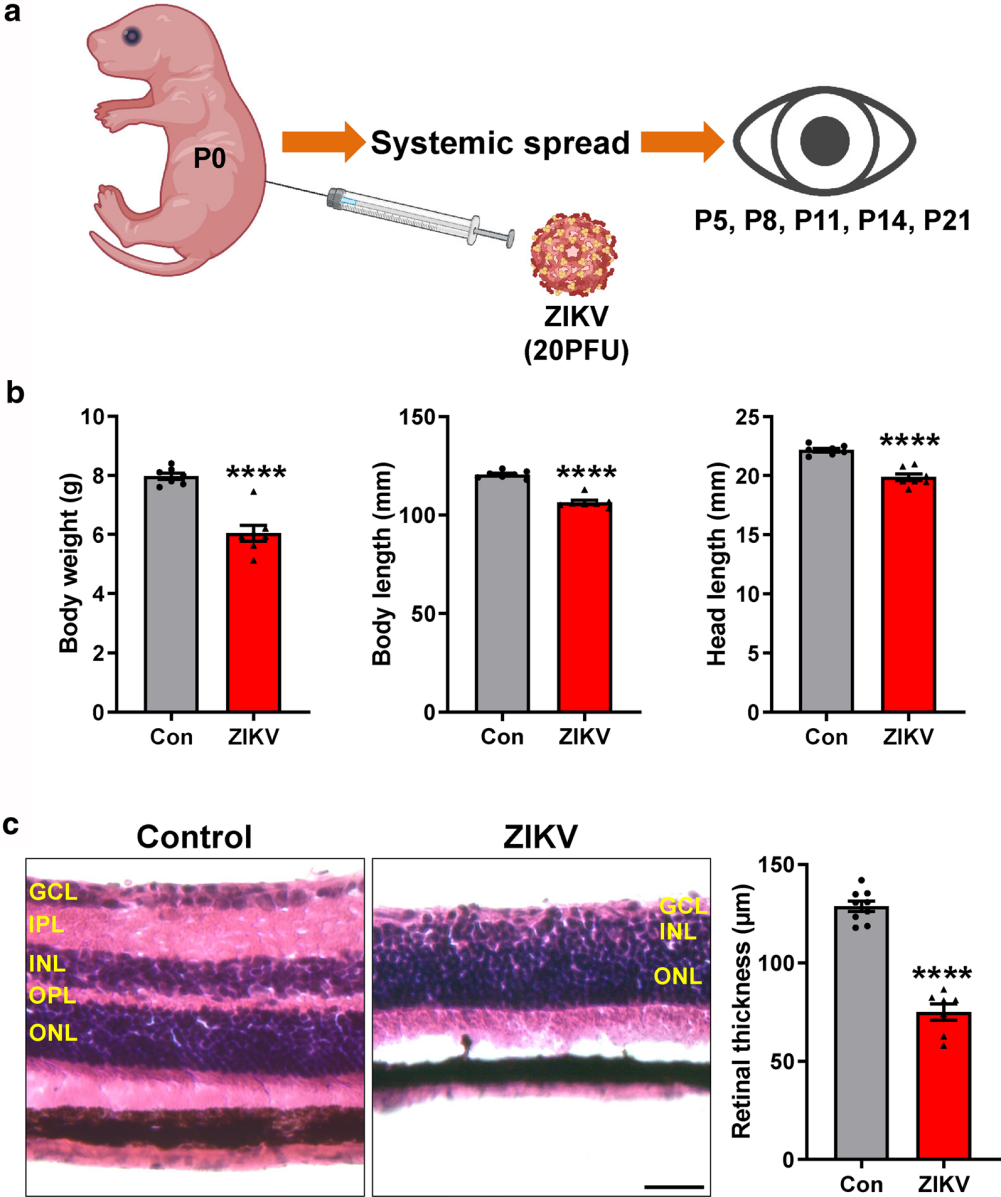
The effects of ZIKV infection on mouse development. **a** Schematic presentation of our model (generated using BioRender). Neonatal C57BL/6 mice were injected with 20 PFU ZIKV at postnatal day (P) 0 subcutaneously. Eyeballs were collected at P5, P8, P11, P14 and P21. **b** Graphs represent body weight, body length including tail and head length of control and ZIKV-infected mice at P21. **c** Representative images of H&E-stained retinal sections from control and ZIKV-infected mice at P21. Graph represents retinal thickness from GCL to ONL. GCL: ganglion cell layer; IPL: inner plexiform layer; INL: inner nuclear layer; OPL: outer plexiform layer; ONL: outer nuclear layer. Scale bar = 50 μm. Data are presented as mean ± S.E.M. n = 7–9 per group. ****p < 0.0001 compared to control mice

To develop a simple and reproducible animal model of ZIKV, we inoculated ZIKV (20 plaque-forming units (PFU)) into neonatal C57BL/6J mice at P0 subcutaneously (Fig. 1a) and observed mouse behavior afterwards until P21. We found that ZIKV-infected mice exhibited less movement, tremors and bilateral hind limb paralysis. They had significantly reduced body weight, body length and head length compared to age-matched uninfected mice (Fig. 1b).

To test whether retinal pathology occurs in mice infected with ZIKV, we stained retinal sections from ZIKV-infected and control mice at P21 by H&E and examined their structures (Fig. 1c). In control group, all retinal layers were intact and noted with dense ganglion cell layer (GCL), inner nuclear layer (INL) and outer nuclear layer (ONL), which were separated by two plexiform layers (inner plexiform layer (IPL) and outer plexiform layer (OPL). However, the retinas of ZIKV-infected mice exhibited greatly reduced total retinal thickness with few GCL cells, attenuated INL and almost disappeared IPL and OPL (Fig. 1c).

**Fig. 2:**
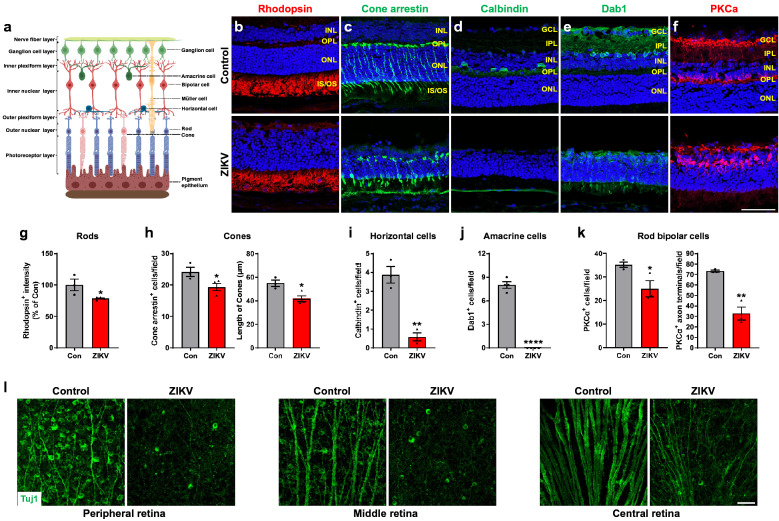
ZIKV infection impairs retinal neurons. **a** Schematic illustration of retinal structure (generated using BioRender). **b**–**f** Immunohistochemical staining on retinal sections from control and ZIKV-infected mice with retinal specific markers, including rhodopsin for rods, cone arrestin for cones, calbindin for horizontal cells, Dab1 for amacrine cells, PKCα for rod bipolar cells at P21. Blue: DAPI staining for nuclei. **g**–**k** Graphs represent the quantification of these cells. **l** Representative images of Tuj1-stained RGCs and their axons in the peripheral, middle and central area of retinal flatmounts at P21. Scale bar = 50 μm. Data are presented as mean ± S.E.M. n = 3–4 per group. *p < 0.05, **p < 0.01, ****p < 0.0001 compared to control mice

To further define the morphological alterations in the retinas of ZIKV-infected mice (Fig. 2a), we performed immunohistochemical staining with antibodies against retinal cell specific markers. In the outer retinas of ZIKV-infected mice, the expression of rhodopsin, the marker for rod photoreceptor cells, showed an apparent decline in immunointensity compared with control retinas (Fig. 2b, g). Moreover, cone photoreceptor cells, evaluated by the staining with antibody against cone arrestin, distributed evenly throughout the retina in control group. In comparison, cone arrestin staining was aberrant, and the number and length of cone photoreceptor cells were significantly decreased in ZIKV-infected retinas (Fig. 2c, h).

We also evaluated alternations of horizontal cells, amacrine cells and rod bipolar cells, which are intermediate neurons connecting photoreceptors and retinal ganglion cells (RGCs), with antibodies against calbindin, Dab1 and PKCα, respectively. We observed that the horizontal cell bodies were located in the INL of control retinas, but they were largely decreased in ZIKV-infected retinas (Fig. 2d, i). Similarly, Dab1, the marker for amacrine cells that contribute to vertical communication within the retina, was expressed in the retinal INL in control mice, whereas it was barely found in ZIKV-infected mice (Fig. 2e, j). Rod bipolar cells are responsible for transmitting signals from photoreceptors to RGCs. In control retina, PKCα was expressed in rod bipolar cell bodies and axon terminals which reach the border between the IPL and GCL. But less rod bipolar cell bodies and axon terminals, and shorter axons were found in ZIKV-infected retinas (Fig. 2f, k).

RGCs are the only output neurons of the retina which collect visual information and send it to the brain through their axons. To further investigate whether RGCs were affected by ZIKV infection, we used Tuj1 antibody, a marker for RGCs in the retina to stain retinal flatmounts from two groups of mice at P21. We found that the number of RGCs and the diameter of axons in the peripheral, middle and central area of the retina were dramatically decreased in ZIKV-infected mice compared to control mice (Fig. 2l). These results indicate all retinal neurons undergo significant loss during ZIKV infection.

**Fig. 3:**
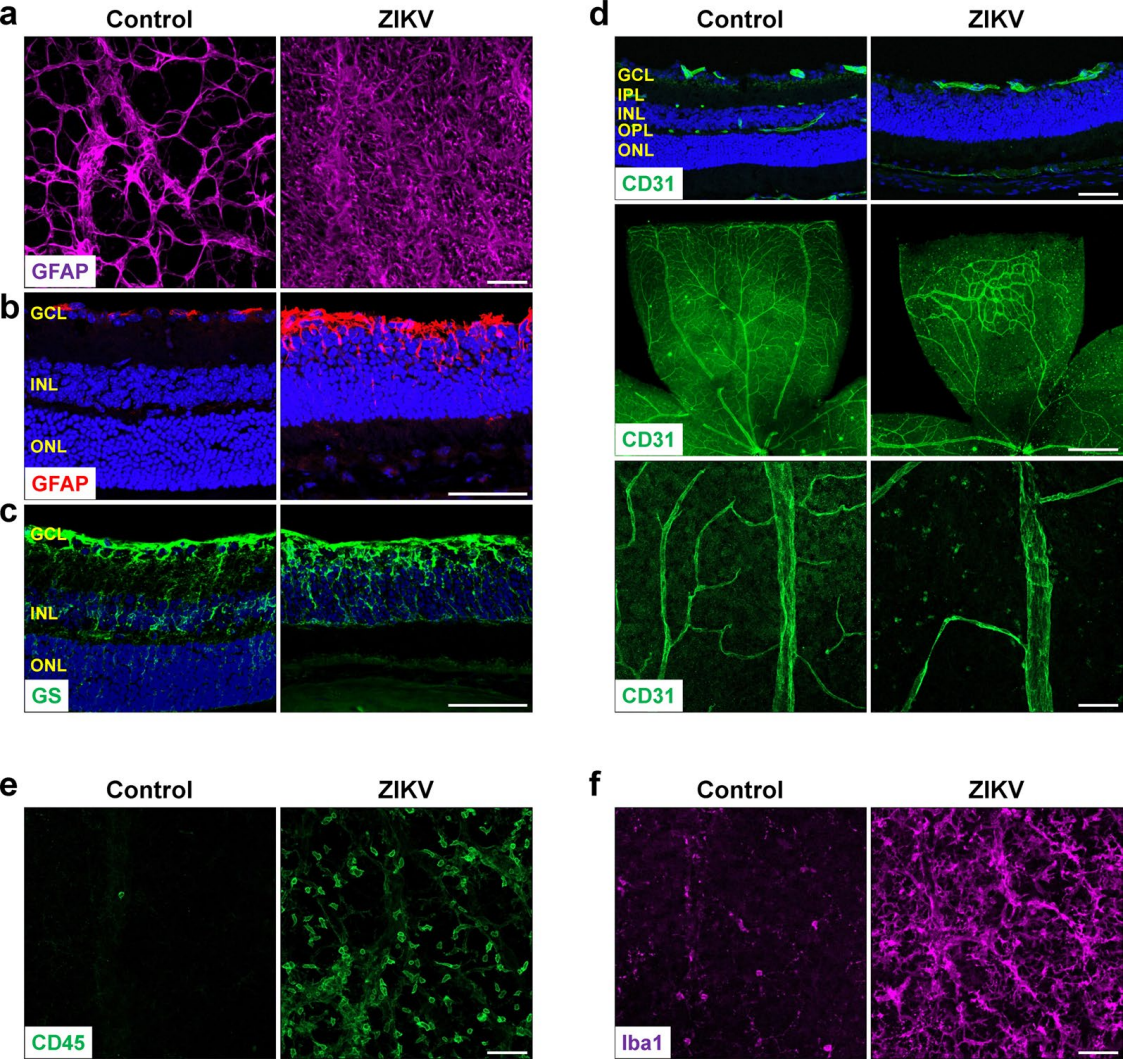
ZIKV infection induces retinal gliosis, vessel impairment and inflammation. ZIKV was injected into neonatal mice at P0 and retinas were collected at P21. **a**, **b** Representative images of GFAP staining in retinal flatmounts and sections. **c** Representative images of glutamine synthetase (GS) staining in retinal sections. **d** CD31 staining for vasculature in retinal sections (upper panel) and flatmounts (middle and lower panels). **e** CD45 staining for leukocytes. **f** Iba1 staining for microglia. Blue: DAPI staining for nuclei. Scale bar = 50 μm except the middle panel in d where scale bar = 500 μm. n = 3–6 per group

Retinal glial cells, which include both Müller cells and astrocytes, are crucial for maintaining normal function of the retina. To assess their changes in the retina after ZIKV infection, we stained retinal flatmounts or sections with antibody against-GFAP, a marker for astrocytes and gliosis, or antibody against-glutamine synthetase (GS), which is expressed in Müller cells and their processes. We observed that the astrocyte network, which serves as a template for angiogenesis and maintains vascular integrity [21], was disorganized and astrocyte gliosis was dramatically increased (Fig. 3a, b). Moreover, GFAP level was dramatically increased in Müller cells (Fig. 3b), which were GS-positive and became shorter with abnormal morphology in ZIKV-infected retinas (Fig. 3c).

These results indicate that ZIKV infection at P0 leads to thinning and aberrant retinal structure and marked loss of retinal neurons, associated with the disruption of astrocyte network and gliosis.

### ZIKV infection elicits loss of retinal vasculature and increase in inflammation

To further characterize the influence of ZIKV on retinal vasculature, we stained ZIKV-infected and control retinas with antibody for endothelial cell marker CD31 at P21. In control mice, the inner retinal vasculature had developed into three layers: the superficial, intermediate and deep layers at P21. In contrast, only the superficial layer of vasculature was observed in retinal section of ZIKV-infected mice (Fig. 3d, upper panel). Moreover, vessel density was greatly decreased while large avascular area was observed in the superficial layer of ZIKV-infected retinas (Fig. 3d, middle and lower panels), suggesting ZIKV infection either inhibits vessel development or induces vessel degeneration.

Immune response and inflammatory cascade play an important role in retinal pathology. Therefore, we further investigated whether ZIKV could cause inflammatory immune response in mice retina. Whole-mount retinas were stained with antibody against CD45, which was expressed on leukocytes. In control group, there were few leukocytes. However, CD45-positive cells were abundant in ZIKV-infected retinas, suggesting ZIKV infection increases leukocyte infiltration in the retina (Fig. 3e). In addition to leukocytes from circulation, microglia play an important role in retinal inflammation by functioning as resident innate immune cells. We subsequently investigated changes of microglia in retinal flatmounts by labeling them with anti-Iba1 antibody. While microglia were sparsely distributed and exhibited a highly ramified morphology (resting state) in control retinas, ZIKV-infected retinas showed massive amount of microglia with retracted processes and amoeboid soma (activated state) [23] (Fig. 3f).

Overall, these data indicate that ZIKV infection can cause a severe vascular phenotype with prominently reduced vascular coverage and vessel density, accompanied with increased inflammation in the retina.

### ZIKV induces progressive retinal degeneration and inflammation

**Fig. 4:**
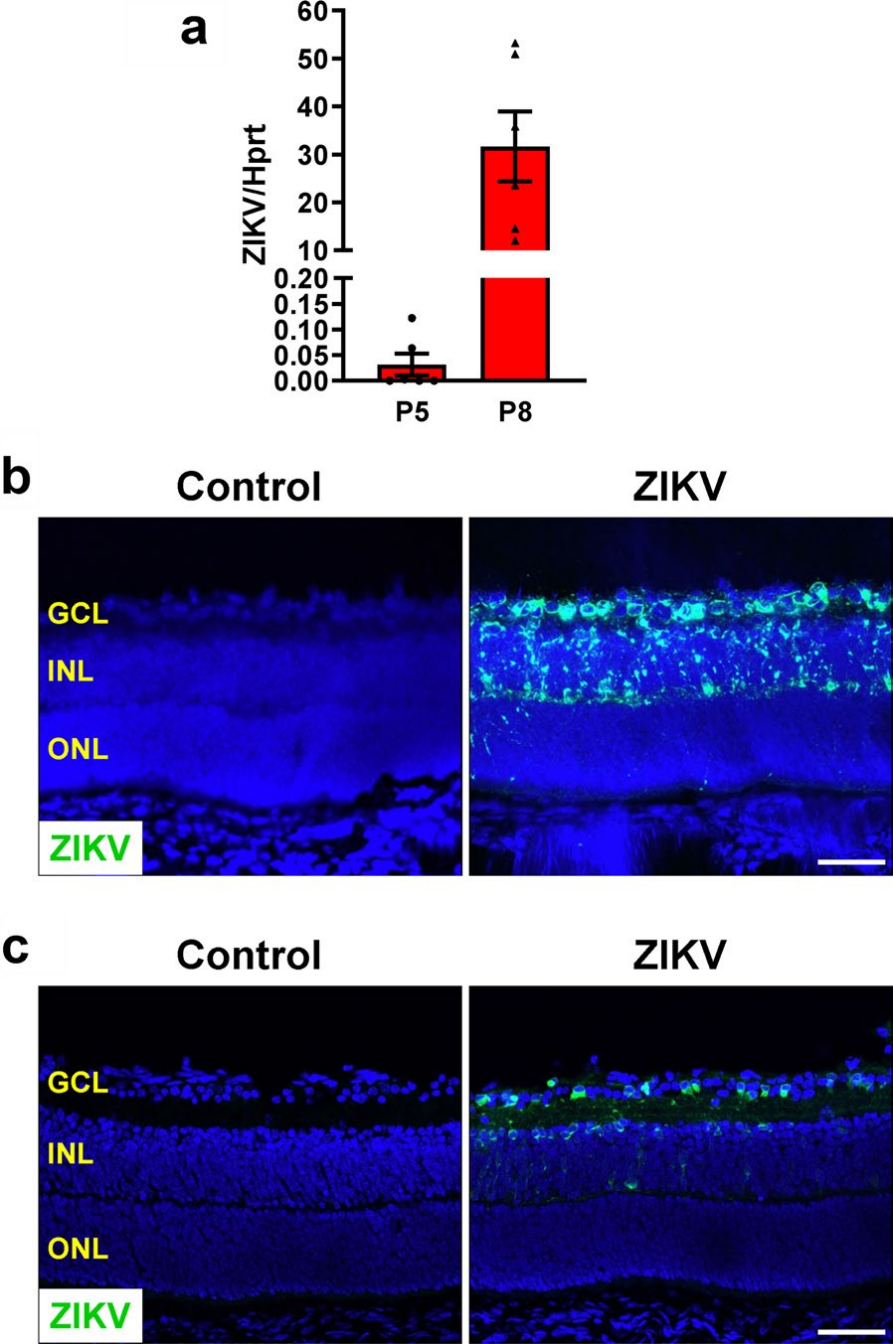
ZIKV expression in the retina. ZIKV was injected into neonatal mice at P0 and retinas were collected at P5 and P8. **a** Viral RNA in the retinas was detected by qPCR. n = 6 per group. **b** Zika viral RNA in retinal sections was assessed by FISH at P8. **c** ZIKV protein expression was examined by immunostaining with a pan flavivirus antibody (4G2) at P8. n = 3 per group

**Fig. 5:**
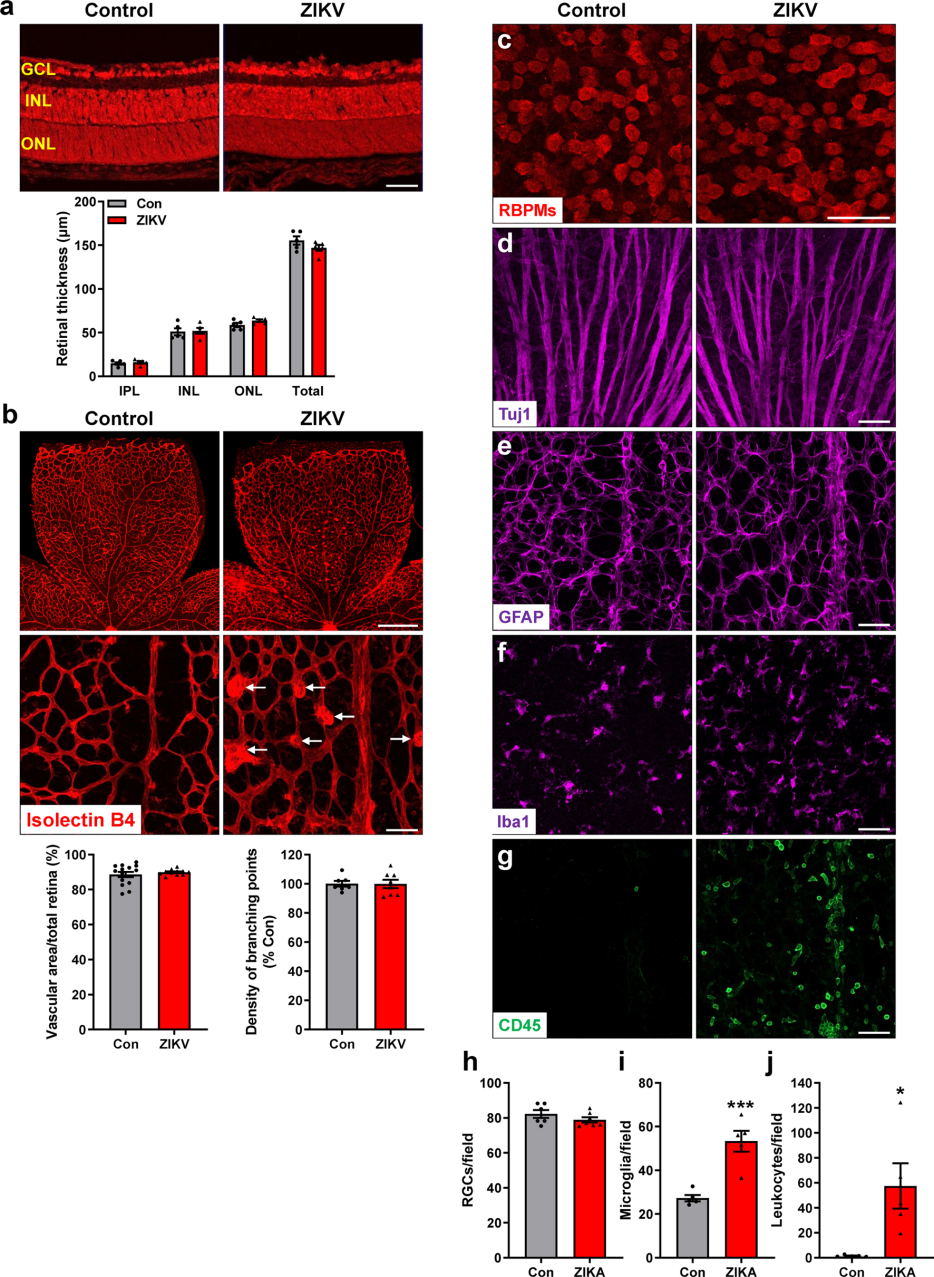
ZIKV induces subtle retinal pathology at P8. ZIKV was injected into neonatal mice at P0 and retinas were collected at P8. **a** Representative images of PI-stained retinal sections at P8. Graph represents the thickness of individual retinal layers and total retina from GCL to ONL. n = 5 per group. **b** Representative images of retinal flatmounts labeled with isolectin B4 for vasculature. Arrows indicate neovascular tufts. Graphs represent vascular area (n = 11–14 per group) and the density of vessel branching points (n = 7–8 per group). **c**–**g** Representative images of retinal flatmounts labeled with anti-RBPMs and Tuj1 for RGCs and their axons, anti-GFAP for astrocytes, anti-Iba1 for microglia, and anti-CD45 for leukocytes. **h**–**j** Graphs represent the quantification of numbers of RGCs (n = 6–7 per group), microglia and leukocytes (n = 5 per group). Scale bar = 50 μm except the upper panel in b where scale bar = 500 μm. *p < 0.05, ***p < 0.001 compared to control mice

To determine the onset of ZIKV-induced retinopathy in our mouse model, we collected samples from ZIKV-infected and control mice at P5, P8, P11 and P14, respectively. ZIKV RNA was detected in 50% of P5 retinas and all P8 retinas, and its average level in P8 retinas was more than 1000-fold higher than that in P5 retinas (Fig. 4a), suggesting P5 was a very early stage when ZIKV entered retinas. Analysis of ZIKV RNA by RNAscope and protein by a pan flavivirus antibody (4G2) revealed that ZIKV-infected cells were mainly present in the GCL and INL at P8 (Fig. 4b, c). Morphologically, at P5, there was no evident difference in retinal thickness (Additional file 1: Fig. S1a), vascular area and vessel density (Additional file 1: Fig. S1b), the number and morphology of RGCs and their axons (Additional file 1: Fig. S1c, S1d and S1g), astrocyte network (Additional file 1: Fig. S1e), and the number of leucocytes (Additional file 1: Fig. S1f and S1h) between ZIKV-infected and control mice. At P8, retinal thickness (Fig. 5a), vascular area and vessel density (Fig. 5b) and astrocyte network (Fig. 5e) were indistinguishable in control and ZIKV-infected retinas. However, a close examination revealed that abnormal neovascular tufts (Fig. 5b, lower panel), increased microglia with activated morphology (Fig. 5f and i) and many leukocytes (CD45^+^ cells) (Fig. 5g and j) were present in ZIKV-infected retinas. Moreover, although no significant difference was observed in the number and morphology of RGCs and their axons (Fig. 5c, d and h), there was a slight trend toward reduced RGCs.

**Fig. 6:**
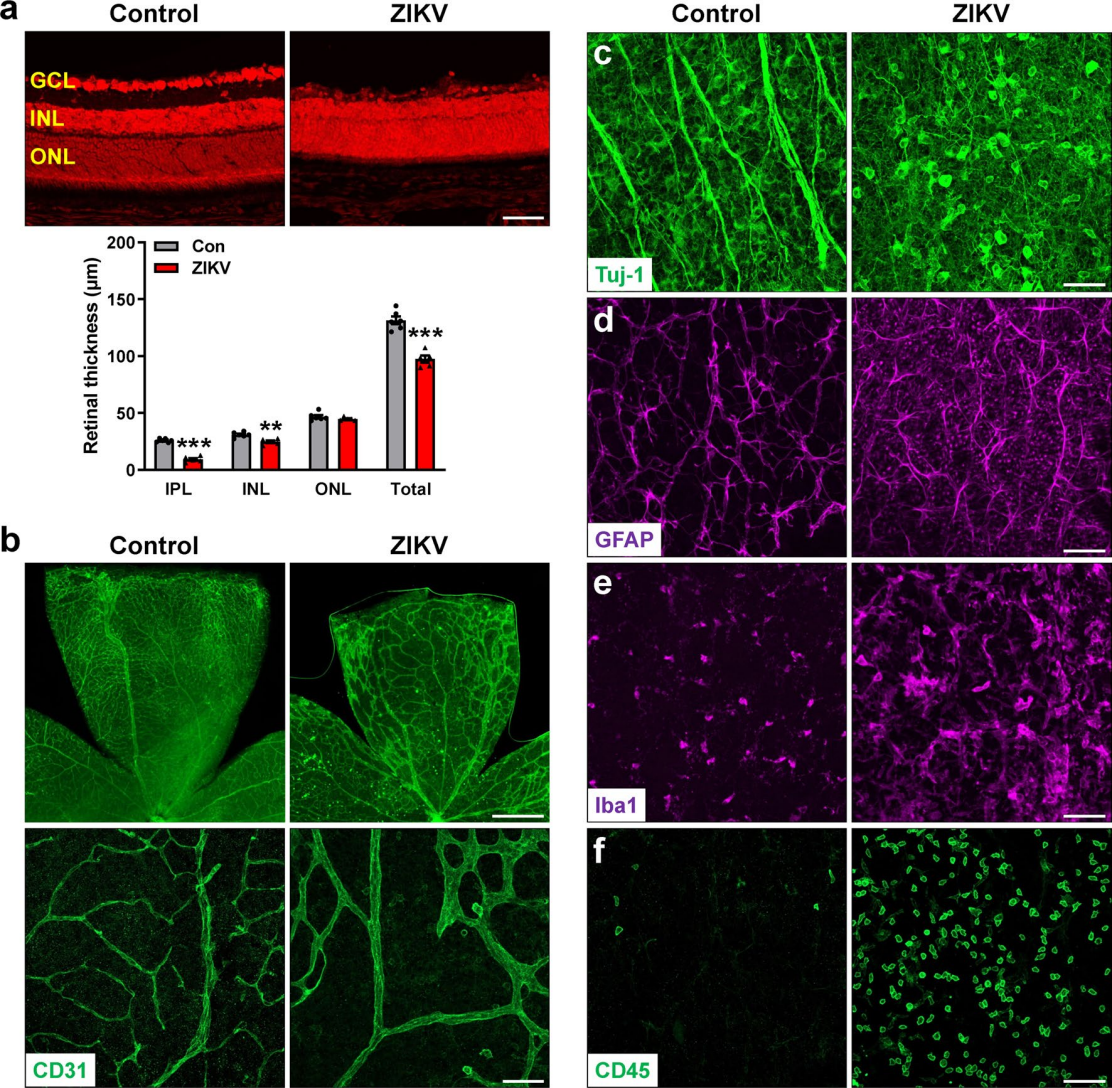
ZIKV further exacerbates retinal degeneration at P14. ZIKV was injected into neonatal mice at P0 and retinas were collected at P14. **a** Representative images of PI-stained retinal sections at P14. Graph represents the thickness of individual retinal layers and total retina from GCL to ONL. **b** Representative images of retinal flatmounts labeled with CD31 for vasculature. **c**–**f** Representative images of retinal flatmounts labeled with anti-Tuj1 for RGCs and their axons, anti-GFAP for astrocytes, anti-Iba1 for microglia, and anti-CD45 for leukocytes. Scale bar = 50 μm except the upper panel in b where scale bar = 500 μm. Data are presented as mean ± S.E.M. n = 5–6 per group. **p < 0.01, ***p < 0.001 compared to control mice

At P11, vessel density was significantly decreased accompanied with microvessel enlargement (Additional file 1: Fig. S2a), and prominent loss of RGC and their axons were observed in ZIKV-infected retinas (Additional file 1: Fig. S2b). As time went on, ZIKV-infected mice turned to be weaker with unsteady gait and mild ataxia at P14. At this time point, retinal morphological changes were much similar to what occurred at P21 (Fig. 6). The thickness of total retina, IPL and INL was all significantly decreased (Fig. 6a). Vascular density was further decreased (Fig. 6b), RGCs and their axons were largely diminished (Fig. 6c) and astrocyte network was disrupted (Fig. 6d), associated with exacerbated microglial recruitment and activation (Fig. 6e) and leukocyte infiltration (Fig. 6f). These results indicate that ZIKV induces progressive retinal degeneration and inflammation from P8 although it does not affect initial vascular development and formation of retinal layers.

### Potential mechanisms of ZIKV-induced retinal degeneration

**Fig. 7:**
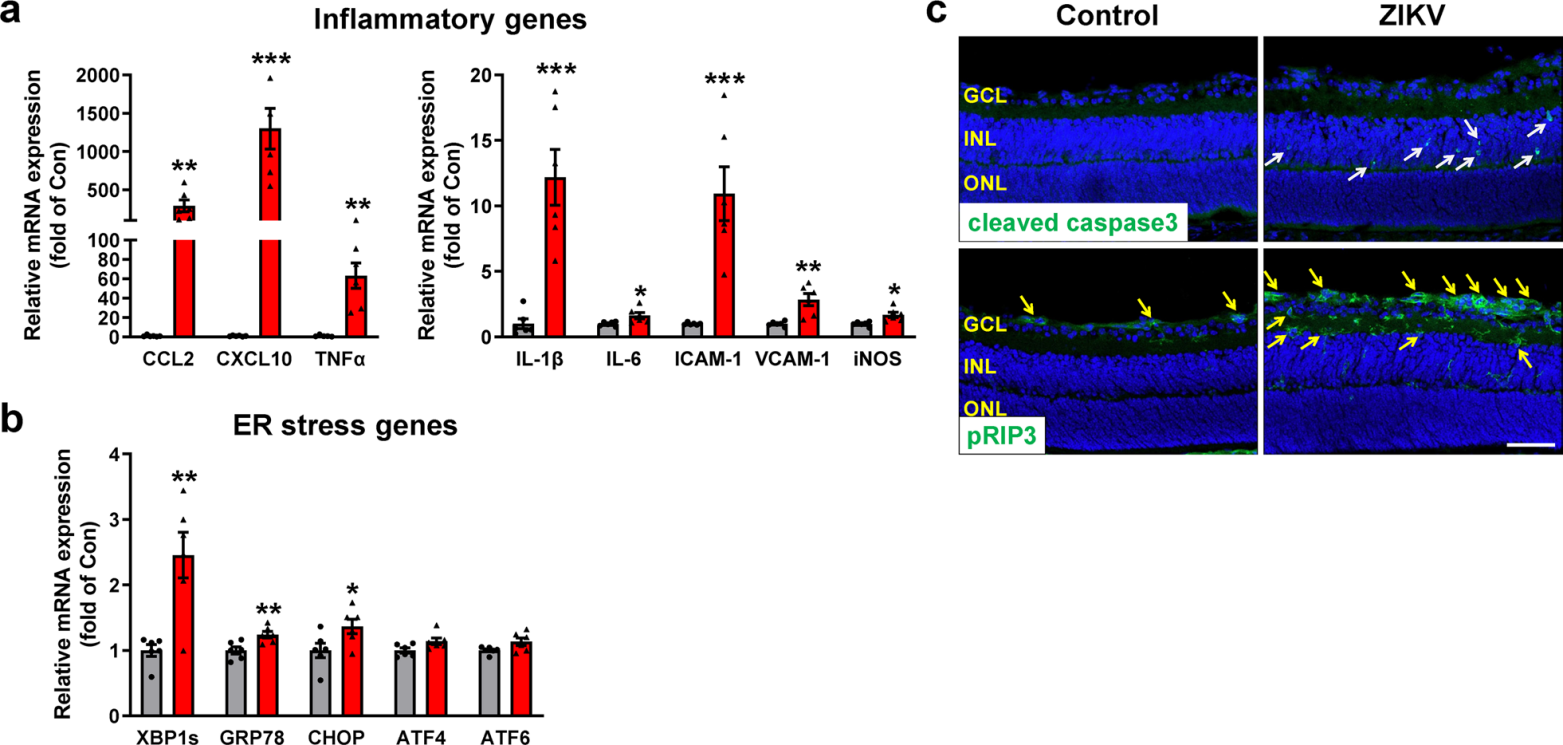
ZIKV infection induces upregulation of inflammatory and ER stress molecules and retinal cell death at P8. ZIKV was injected into neonatal mice at P0 and retinas were collected at P8. **a**, **b** Retinal RNA was extracted and analyzed by qPCR for the expression of inflammatory genes and ER stress genes. n = 5–6 per group. **c** Immunostaining with antibodies against cleaved caspase3 (upper panel) and pRIP3 (lower panel) in retinal sections of control and ZIKV-infected mice. Blue: DAPI staining for nuclei. Arrows indicate apoptotic or necroptotic cells. Scale bar = 50 μm. n = 3–4 per group. Data are presented as mean ± S.E.M. *p < 0.05 **p < 0.01, ***p < 0.001 compared to control mice

Excessive or uncontrolled inflammation and endoplasmic reticulum (ER) stress are key mediators for cell death [13]. To determine the potential mechanisms of ZIKV-induced retinal degeneration, we analyzed retinal inflammation and ER stress in P8 retinas (Fig. 7a and b). We found that levels of several key inflammatory molecules including chemokines (CCL2 and CXCL10), pro-inflammatory cytokines (TNFα, IL-1β and IL-6), adhesion molecules (ICAM-1 and VCAM-1) and iNOS were all increased 1.6–1300 folds in ZIKV-infected retinas. Similarly, key molecules involved in ER stress, including XBP1s, GRP78 and CHOP, were significantly increased. Associated with increases in inflammation and ER stress, analysis of cleaved caspase 3, a marker for apoptosis, and phosphorylated receptor-interacting protein 3 (pRIP3), a marker for necroptosis, revealed that both cleaved caspase 3 and pRIP3 were significantly increased in ZIKV-infected retinas in which cleaved caspase 3-positive cells were mainly localized in the INL whereas pRIP3-positive cells were mainly located in the GCL and IPL (Fig. 7c). These results suggest that ZIKV-induced retinal degeneration may involve inflammation and ER stress-mediated cell apoptosis and necroptosis.

### Evaluation of the drug efficacy in the model of ZIKV-induced retinopathy

**Fig. 8:**
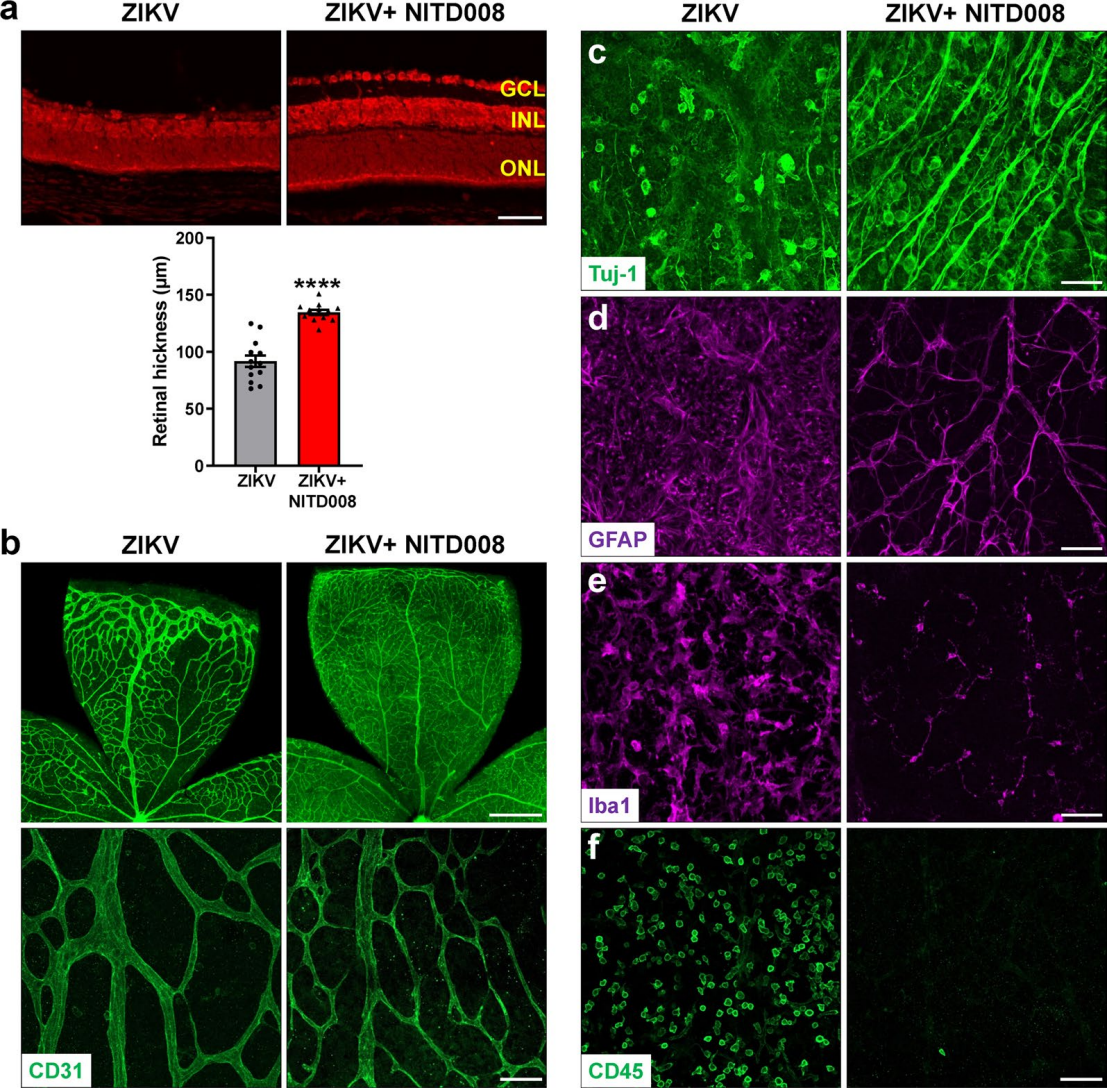
NITD008 treatment blocks ZIKV-induced retinal abnormalities. ZIKV was injected into neonatal mice at P0, followed by NITD008 treatment right after ZIKV infection and every other day until day 12, and retinas were collected at P14. **a** Representative images of PI-stained retinal sections at P14. Graph represents the thickness of total retina from GCL to ONL. n = 12–13 per group. **b** Representative images of retinal flatmounts labeled with CD31 (green) for vasculature. **c**–**f** Representative images of retinal flatmounts labeled with anti-Tuj1 for RGCs, anti-GFAP for astrocytes, anti-Iba1 for microglia, and anti-CD45 for leukocytes. n = 4–8 per group. Scale bar = 50 μm except the upper panel in b where scale bar = 500 μm. Data are presented as mean ± S.E.M. ****p < 0.0001 compared to control mice

Having established and characterized this mouse model of ZIKV-induced retinopathy, we assessed whether it could be used for drug discovery. Recent studies have showed that antiviral agent sofosbuvir (SOF; a clinically approved drug for hepatitis C virus) could reduce ZIKV viral load [2, 10], therefore we tested if SOF can inhibit ZIKV-induced retinal degeneration. P0 pups were infected with ZIKV, followed by administration of SOF (80 mg/kg) or vehicle control every day until P13. At P14 when ZIKV-induced retinal degeneration was evident, we examined ZIKV-induced retinal alternations. We found that there was significant variation in SOF-treated pups in which SOF only successfully blocked ZIKV-induced retinal degeneration in 44% pups (Table 1 and Additional file 1: Fig. S3), suggesting SOF is not a sufficient inhibitor for ZIKV infection. NITD008 is a selective flavivirus inhibitor that shows potent efficacy against ZIKV infection [7, 35]. We administered NITD008 (an adenosine analog, 5 mg/kg) or vehicle control right after ZIKV injection and once every other day until day 12, and found that all NITD008-treated ZIKV-infected mice exhibited normal retinal structure which was comparable with non-infected mice (Table 1 and Fig. 8a). Moreover, NITD008 treatment blocked ZIKV-induced degeneration of retinal vasculature (Fig. 8b) and RGCs (Fig. 8c), and gliosis and inflammation (Fig. 8d–f). Collectively, these results indicate that our model can be used to evaluate drug efficacy and NITD008 is an effective candidate to treat ZIKV-induced retinopathy.

**Table 1:** Comparison of the efficacy of SOF and NITD008 against ZIKV-induced retinal pathology

Inhibitor	ZIKV-infected mice with inhibitor	Mice with normal retinas	Inhibition rate (%)
SOF	9	4	44
NITD008	12	12	100

ZIKV was injected into neonatal mice at P0, followed by SOF or NITD008 treatment, and retinas were collected at P14 and compared for abnormalities

### Evaluation of the safety of ZIKV vaccine candidate

**Fig. 9:**
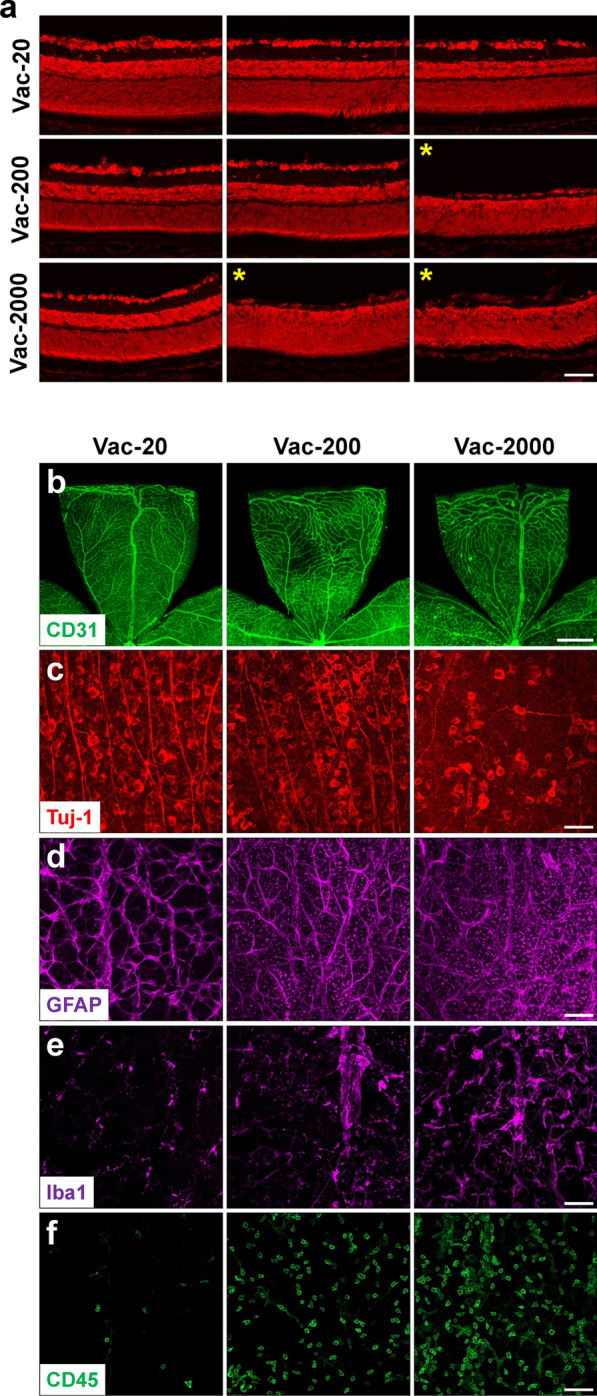
The effects of live attenuated ZIKV vaccine candidate on retina. Various dose of a live-attenuated ZIKV vaccine candidate (Vac-20 or 200 or 2000 PFU) was subcutaneously injected into neonatal mice at P0 and retinas were collected at P21. **a** Representative images of PI-stained retinal sections. Stars indicate retinal samples with decreased thickness. **b**–**f** Representative images of retinal flatmounts labeled with anti-CD31 for vessels, anti-Tuj1 for RGCs and their axons, anti-GFAP for astrocytes, anti-Iba1 for microglia, and anti-CD45 for leukocytes. Scale bar = 50 μm except b where scale bar = 500 μm. n = 5 for Vac-20; n = 8 for Vac-200 and Vac-2000

There is currently no licensed human vaccine for ZIKV, but several vaccine candidates have been developed and evaluated in preclinical and clinical studies [27]. We tested whether our mouse model can be used to evaluate the safely of ZIKV vaccine candidates. We chose to evaluate a live-attenuated ZIKV vaccine with a 3’UTR deletion (10-del ZIKV) [25] in the current model. After subcutaneously inoculating the live-attenuated ZIKV vaccine (20, 200, or 2000 PFU) into neonatal C57BL/6 mice at P0 and collecting retinas at P21, we found the low dose had no effect on retinal structure, but medium and high doses of vaccine candidate caused retinal pathology at rates of 50% and 87.5% respectively (Table 2). The retinas with abnormality exhibited decreased retinal thickness (Fig. 9a), reduced vasculature (Fig. 9b), loss of RGCs (Fig. 9c), and/or increased gliosis (Fig. 9d) and microglial activation and leukocyte infiltration (Fig. 9e and f) as seen in ZIKV-infected WT retinas. These data suggest that this live-attenuated ZIKV vaccine may potentially induce retinal degeneration if non-optimal dose was provided during pregnancy.

**Table 2:** The abnormal rate of the retinas after injection with ZIKV vaccine candidate

ZIKV-vaccine dose (PFU)	Total vaccinated mice	Mice with abnormal retina	Abnormal rate (%)
20	5	0	0
200	8	4	50
2000	8	7	87.5

Various dose of a live-attenuated ZIKV vaccine candidate was injected into neonatal mice at P0, and retinas were collected at P21 and compared for abnormalities

## Discussion

Mouse models have been widely used in biomedical research due to low-cost maintenance, a short reproductive cycle and easy genetic modification. In this study, we developed a novel and reproducible model of ZIKV-induced retinopathy using immunocompetent C57BL/6 mice. We demonstrated that P0 pups inoculated with ZIKV developed significant loss of RGCs and their axons, and retinal thinning during growing-up, which recapitulates a series of clinical features reflecting retinal neuronal and axonal degeneration when using non-invasive imaging to examine infants with Congenital Zika Syndrome, including neurosensory retinal thinning, discontinuation of the photoreceptor inner and outer segment junction, and optic nerve hypoplasia [33]. Moreover, retinal pathologic changes and the severity of retinal neuronal degeneration in our model are comparable to those when mice are infected at E13.5 and E15 via intra-amniotic injection with ZIKV [6, 29] though it is much easier to infect neonatal mice by subcutaneous injection of ZIKV, which brings virus to the retina via blood circulation, similar to what occurs following intra-amniotic injection. Clinically, among completed pregnancies with positive nucleic acid tests confirming ZIKV infection identified in the first, second, and third trimesters, CDC’s analysis demonstrated the percentage of fetuses or infants with possible Zika-associated birth defects was 8%, 5%, and 4%, respectively [28]; another study of pregnancy outcomes after ZIKV infection in French Territories in the Americas also found that neurologic and ocular defects were 12.7%, 3.6% and 5.3%, respectively [14]. Therefore, our model is disease-relevant since pups are infected with ZIKV at a time point equivalent to human retinal development in the third trimester when ZIKV infection could still cause birth defects.

In addition to neurodegeneration, we found dramatic decreases of retinal vascular coverage and density in ZIKV-infected retinas. While ZIKV-induced neuronal damage is well appreciated in the brain and retina, the impact of ZIKV on vessels is largely unknown although a few in vitro studies demonstrate ZIKV could infect and replicate in endothelial cells, break down endothelial barriers and impair endothelial cell lipid homeostasis [17, 20, 22, 37], and Garcez et al. reported reduction in the vasculature density and vessel branching in the brain from IFN-deficient mouse with congenital ZIKV infection and reduced retinal vascular network was displayed in one image of this article [12]. Our study represents the first one to investigate ZIKV-induced retinal vasculature changes in detail. Unlike Garcez et al.’s study suggesting that the reduction of vasculature is caused by perturbation of vascular development by ZIKV [12], we found that initial retinal vascular development at P5 and P8 after ZIKV inoculation was not retarded although vascular tufts were noticed at P8. From P11 to P21, progressive reduction of vascular network was observed and large avascular area in the retina was found at P21, associated with extensive alterations in astrocyte morphology and disruption of its network that is usually in line with vasculature and supports vessels. These data suggest that ZIKV indeed induces significant retinal vascular degeneration in addition to possible perturbation of vascular development.

Of note, significant changes in vasculature are also found in infants with congenital Zika syndrome. In one study using OCT to examine retinal abnormalities, vessels appear very sparse and thin in some fundus photography images [33]. In another study using fluorescein angiography to evaluate retinal vasculature changes, diffuse avascularity of the peripheral retina, retinal vessels rectification, abnormal arteriovenous shunts, and vascular attenuation, leakage and tuft are observed [32]. These observations further support that our model is clinically relevant.

Using this model, we performed studies with drug and vaccine discovery. We evaluated the therapeutic effects of two potential ZIKV antiviral compounds. While the administration of sofosbuvir at 20–33 mg/kg/day was shown to increase survival rate and prevent body weight loss in Swiss mice infected with ZIKV (i.p., 2 × 10^7^ PFU) at P3 [10] and in anti-IFNAR1 antibody-treated C57BL/6J mice infected with ZIKV (s.c., 10^5^ PFU) at 5 weeks of age [2], sofosbuvir at 80 mg/kg/day in our model only exhibited protection in a portion of mice. In contrast, adenosine analog NITD008 which exhibits potential antiviral activity against several Flaviviruses including ZIKV [7] potently prevented ZIKV-induced retinal neuronal and vascular degeneration and inflammation at 5 mg/kg once every other day. The different effects between NITD008 and sofosbuvir are consistent with their in vitro efficacies in which NITD008 inhibits ZIKV in Vero cells with an IC50 of less than 1 µM whereas sofosbuvir inhibits ZIKV in Huh7 cells with an IC50 around 4 µM but has no effects in Vero and A549 cells even at 50 µM [19]. Therefore NITD008 is a more effective candidate than sofosbuvir against ZIKV-induced retinal pathology and warrants to further investigate the efficacy of this compound against ZIKV in pre-clinical and clinical studies. In addition, we investigated the safety of a live-attenuated ZIKV vaccine candidate (10-del ZIKV) [25]. While 10-del ZIKV does not cause mortality when it is intracranially inoculated into P1 CD-1 mice at 1000 times of lethal infectious focus units of wild-type ZIKV [25], it induced retinal abnormality in this model though at much attenuated effects compared to wild-type ZIKV. Therefore, precautions and further optimization of the candidate vaccine are needed to achieve better safety when it is given to pregnant women. Altogether, these studies indicate this model is a sensitive one for drug and vaccine discovery. It has clinically relevant features and can provide additional information which other models may have overlooked.

At present, the mechanisms underlying ZIKV-induced retinal degeneration remains to be elucidated. By analyzing retinal changes at different time points after ZIKV infection, we found retinal neural and vascular development appeared normal at P5 and P8 but expressions of genes involved in inflammation and ER stress were significantly upregulated in P8 retinas of ZIKV-infected pups. Considering inflammation and ER stress play key roles in cell death, it is possible that they are partially involved in ZIKV-induced increases in retinal cell apoptosis and necroptosis at P8 and subsequent progress of retinal degeneration from P11 to P21. Further studies to modulate these two pathways and determine their effects on ZIKV-induced retinopathy are needed to test this possibility. Moreover, an integrated approach with single-cell and omics technologies would often provide better insights on this process. One advantage of our model is that it is easier to study molecular mechanisms with genetically modified mice than the model with intra-amniotic injection since transgenic mice often have lower birth rates than WT mice and ZIKV infection during pregnancy could further reduce it [6, 29].

## Conclusions

In summary, we developed an easily inducible, reproducible and clinic-relevant mouse model of ZIKV-induced retinopathy. Using this model, we provided the first evidence that retinal vessels similar to neurons underwent progressive degeneration after ZIKV infection, and inflammation, ER stress, apoptosis and necroptosis may be potentially involved in this process. Moreover, we demonstrated the feasibility of using this model in drug discovery and vaccine safety evaluation and found that NITD008 is a better drug candidate than sofosbuvir in treating ZIKV-induced retinopathy.

## Supplementary Information

**Additional file 1:** Supplementary Figures.

## Data Availability

All data generated or analyzed during this study are included in this published article and its supplementary information files.

## Abbreviations

cDNA: Complementary DNA
ddH_2_O: Double distilled water
E: Embryonic day
ER: Endoplasmic reticulum
FISH: Fluorescence in situ hybridization
GCL: Ganglion cell layer
GS: Glutamine synthetase
H&E: Hematoxylin and Eosin
IFNAR1: Type I interferon receptor
INL: Inner nuclear layer
IPL: Inner plexiform layer
ONL: Outer nuclear layer
OPL: Outer plexiform layer
P: Postnatal day
PFA: Paraformaldehyde
PFU: Plaque-forming units
pRIP3: Phosphorylated receptor-interacting protein 3
RGCs: Retinal ganglion cells
SOF: Sofosbuvir
WT: Wild type
ZIKV: Zika virus
