# Supplementary material for: Zika virus induces neuronal and vascular degeneration in developing mouse retina
Source: Acta Neuropathol Commun. 2021 May 25;9:97. doi: 10.1186/s40478-021-01195-6 (PMC8147371; doi:10.1186/s40478-021-01195-6)
Supplement: Supplementary file 1 — Additional file 1: Supplementary Figures. [file 40478_2021_1195_MOESM1_ESM.pdf]

Supplementary Figure 1

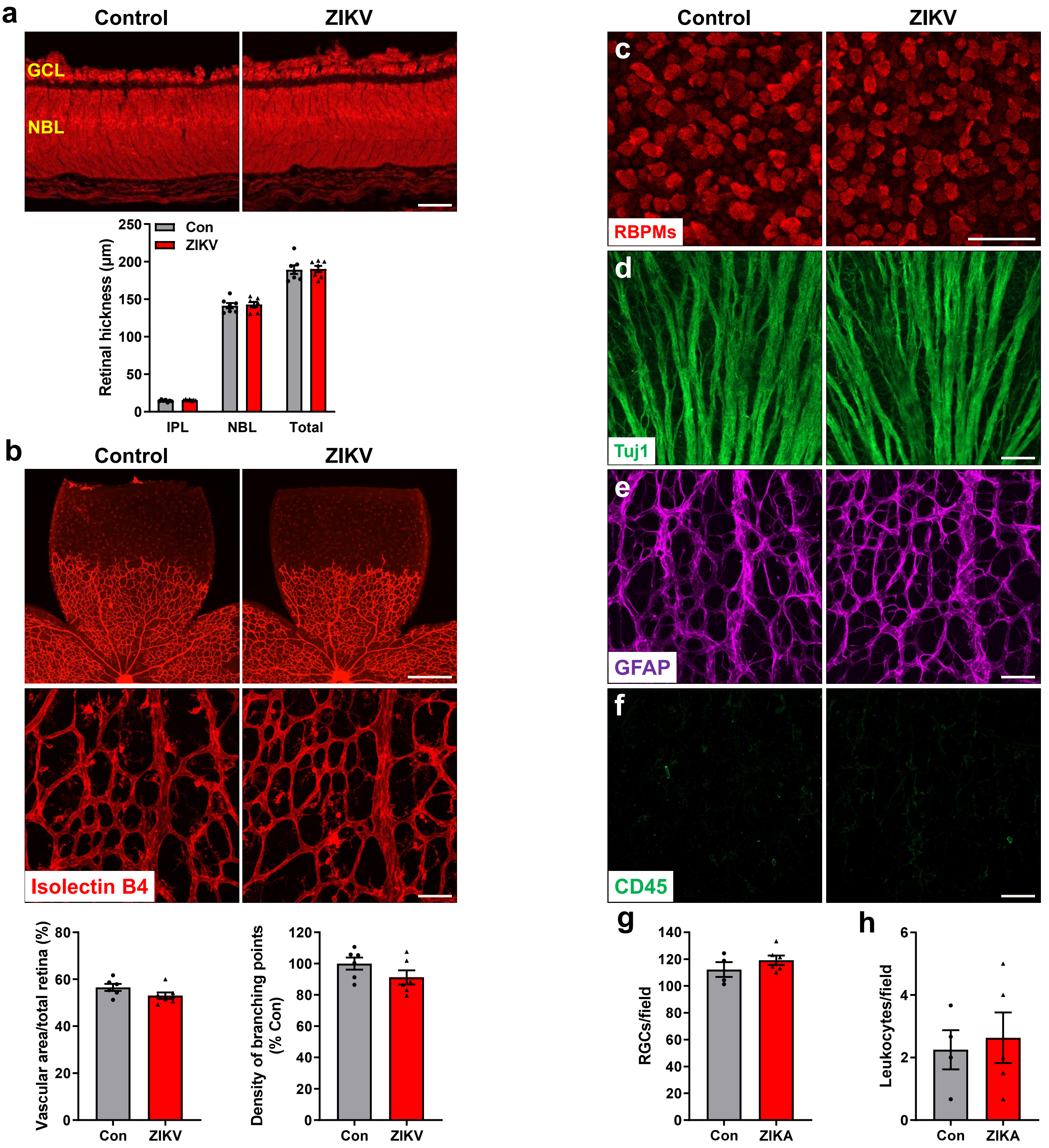

Supplementary Figure 1. ZIKV does not impact the retina at P5. ZIKV was injected into neonatal mice at P0 and retinas were collected at P5. (a) Representative images of PI-stained retinal sections at P5. Graph represents the thickness of individual retinal layers and total retina from GCL to NBL. (b) Representative images of retinal flatmounts labeled with isolectin B4 for vasculature. Graphs represent vascular area and the density of vessel branching. (c-f) Representative images of retinal flatmounts labeled with anti-RBPMs and Tuj1 for RGCs and their axons, anti-GFAP for astrocytes, and anti-CD45 for leukocytes. (g-h) Graphs represent the quantification of numbers of RGCs and leukocytes. Scale bar=50 μm except the upper panel in b where scale bar=500 μm. Data are presented as mean± S.E.M. n=4-7 per group. GCL: ganglion cell layer; NBL: neuroblast layer.

Supplementary Figure 2

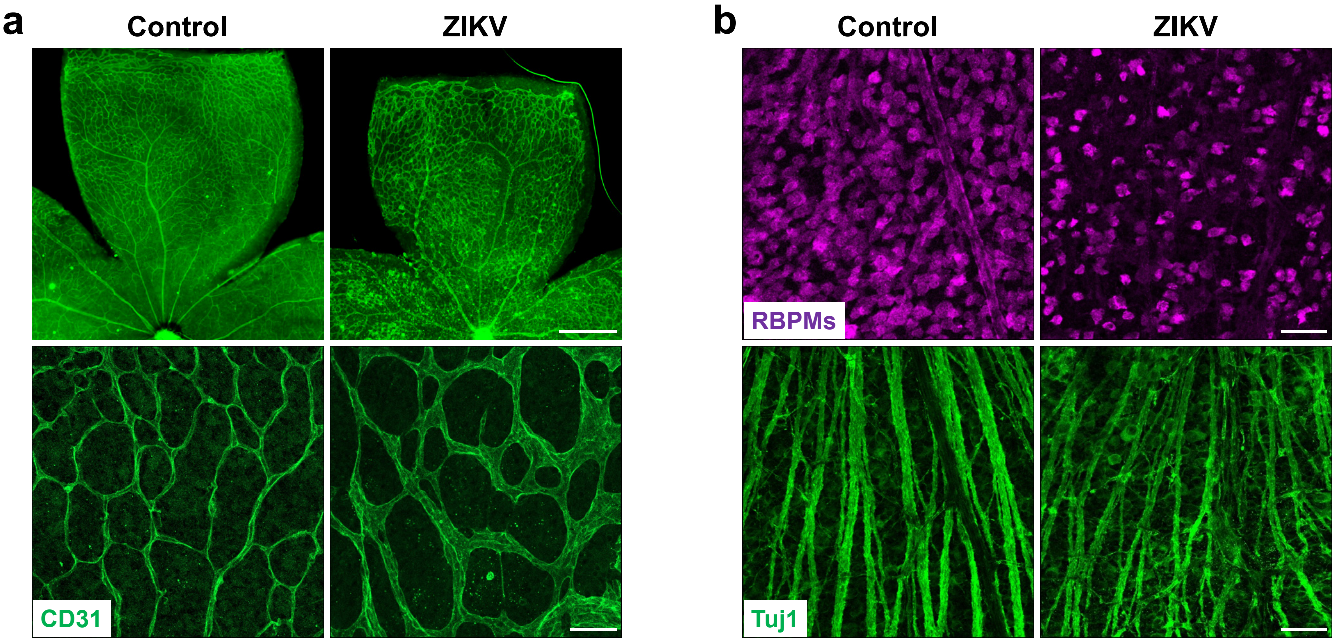

Supplementary Figure 2. ZIKV induces retinal pathology at P11. ZIKV was injected into neonatal mice at P0 and retinas were collected at P11. (a) Representative images of retinal flatmounts labeled with CD31 for vasculature. (b) Representative images of retinal flatmounts labeled with anti-RBPMs and Tuj1 for RGCs and their axons. Scale bar=50 μm except the upper panel in a where scale bar=500 μm. n=5-9 per group.

Supplementary Figure 3

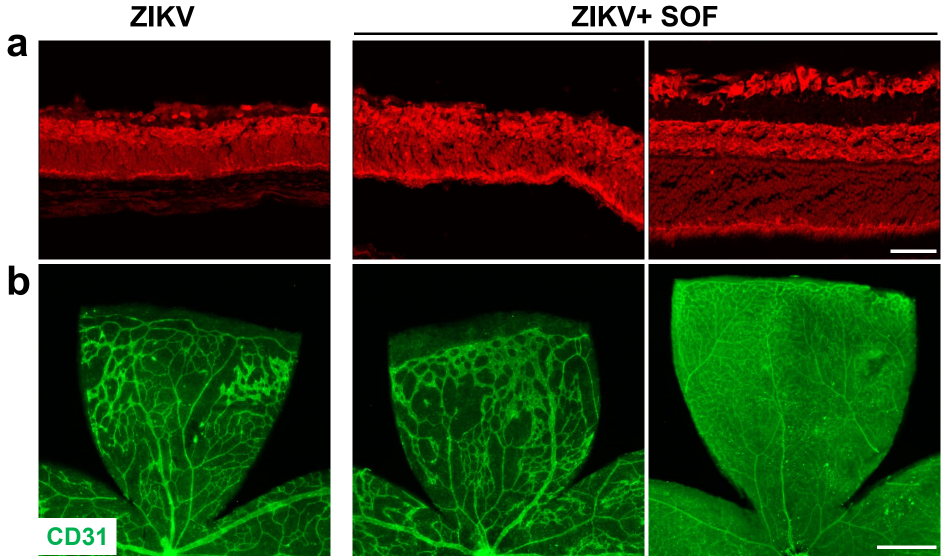

Supplementary Figure 3. Sofosbuvir (SOF) treatment only partially blocks ZIKV-induced retinopathy. ZIKV was injected into neonatal mice at P0, followed by SOF treatment right after ZIKV infection and every day until day 13, and retinas were collected at P14. (a) Representative images of PI-stained retinal sections at P14. Scale bar=50 μm. (b) Representative images of retinal flatmounts labeled with CD31 for vasculature. Scale bar=500 μm. n=5-9 per group.
